# TAP-Stenting Technique for Bifurcation Stenosis of Celiac Artery

**DOI:** 10.1155/2015/468561

**Published:** 2015-02-26

**Authors:** Yucel Colkesen, Taner Seker, Osman Kuloglu, Murat Çayli

**Affiliations:** Department of Cardiology, Adana Numune Hospital, Seyhan, 01140 Adana, Turkey

## Abstract

We report a clinical course of a patient who developed severe ischemic liver injury and total occlusion of the celiac artery (CA). A 40-year-old man presented with abdominal pain. Computed tomography indicated total occlusion of the CA. Laboratory data demonstrated markedly elevated hepatic enzymes. An exploratory laparotomy was not necessitated due to absence of peritonism. The patient was successfully treated by endovascular recanalization of the CA occlusion via transcatheter balloon angioplasty and TAP-stenting (T-stenting and small protrusion) technique. Endovascular intervention in patients solely with liver failure appears practicable and early treatment is advised.

## 1. Introduction

Endovascular intervention (EI) is a practicable option for both acute and chronic mesenteric ischemia (MI). EI includes various techniques, such as catheter-directed vasodilation, thrombus aspiration, thrombolytic therapy, angioplasty, and stenting. The benefits comprise timely visualisation of the affected vascular anatomy with prompt recovery of flow and substitution of open surgical reconstruction [1]. Successful endovascular treatment is associated with improved mortality compared with traditional therapy [2].

The present report describes a case of total occlusion of the celiac artery (CA) resulting in liver injury. Ischemic liver injury instead of bowel ischemia was the vital disturbance that formed the course of the intervention. He was successfully treated by endovascular recanalization of the CA occlusion.

## 2. Case Presentation

**Figure 1:**
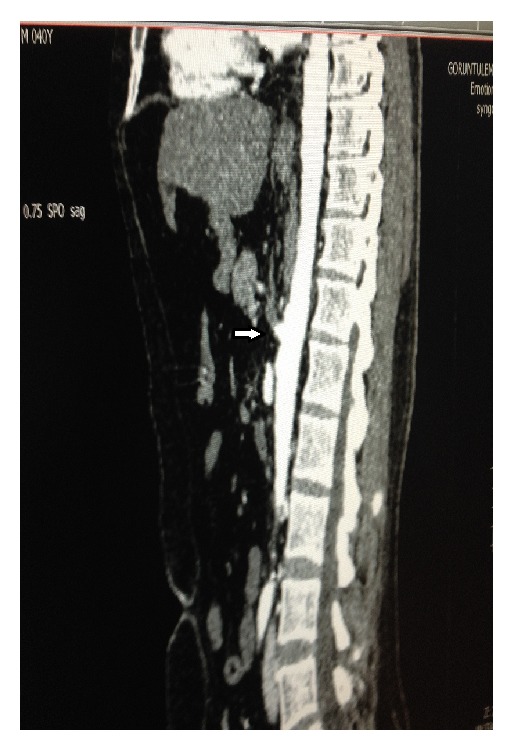
Lateral reconstruction of a contrast-enhanced CT of the abdomen demonstrates occlusion of the CA with visible stump (arrow).

A 40-year-old man presented with a history of liver failure and sudden onset of abdominal pain. He was hospitalized at the department of general surgery. The patient's medical history was unremarkable. On physical examination, he had absence of signs of peritonism. Thus, an exploratory laparotomy was not necessitated. Laboratory data revealed leukocytosis (23900 × 10^3^ *μ*L^−1^) and elevated levels of amylase (124 U/L), aspartate aminotransferase (3145 IU/L), alanine aminotransferase (2945 IU/L), creatinine kinase (1561 IU/L), total bilirubin (1.2 mg/dL), international normalised ratio (2.1), and activated partial thromboplastin time (25 sec). An urgent computed tomography angiography revealed CA occlusion (Figure 1). Superior and inferior mesenteric arteries were intact. Surgeons requested an aortogram and recanalization, if applicable.

**Figure 2:**
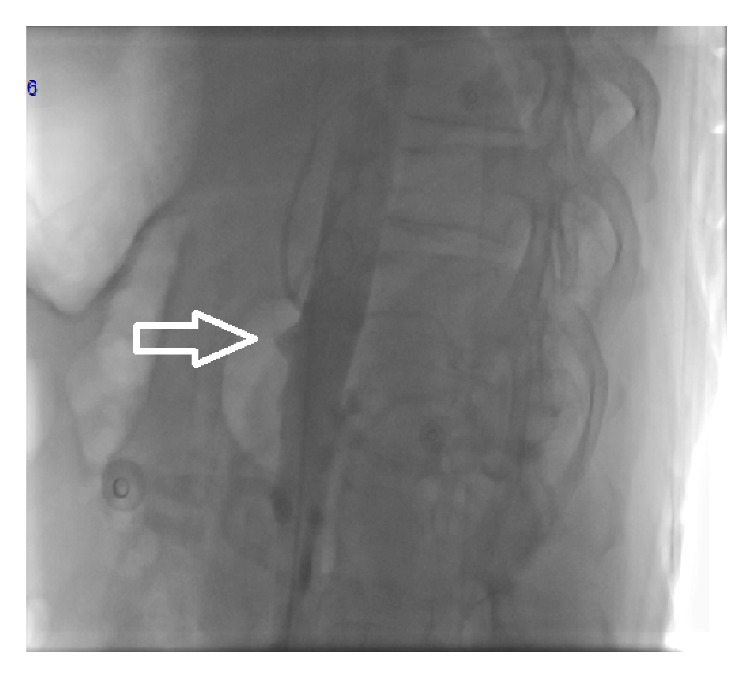
Angiography in a lateral view showing the stump of the CA (arrow).

**Figure 3:**
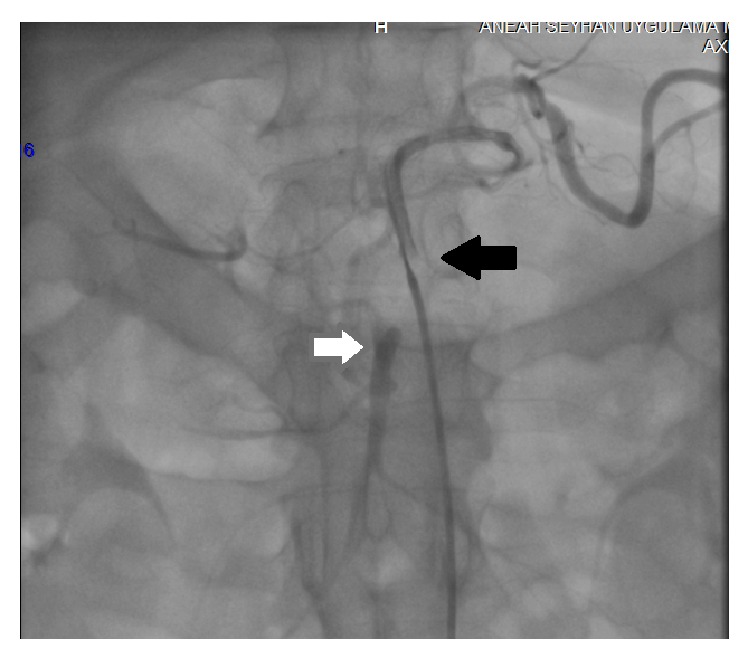
Flows in CA, SA (black arrow), and SMA (white arrow) following deployment of balloon expandable stent.

Anterioposterior and lateral aortograms were performed via the transfemoral approach. The aortogram demonstrated total occlusion of the CA with a visual stump (Figure 2). Retrograde and antegrade opacification of superior mesenteric artery and antegrade flow in inferior mesenteric artery were imagined (Figure 3). A bolus of 10.000 IU intravenous heparin and 600 mg loading dose of clopidogrel were administered immediately after the decision was taken for recanalization.

A 7-French (Fr) sheath from the right femoral artery was inserted. A 7-Fr Judkins right guiding catheter (JR3.5, Medtronic, MN, USA) was used to select the origin of the CA. The total occlusion could not be crossed with a 0.014-inch floppy guidewire (Asahi Soft, Asahi Intecc, Aichi, Japan) at first attempt. Then a 0.014-inch stiff guidewire (Asahi Standart, Asahi Intecc, Aichi, Japan) was employed to cross the lesion. The occlusion could be crossed towards splenic artery (SA). A faint antegrade flow was observed beyond the occlusion, with incomplete filling of the distal bed. Median arcuate ligament syndrome was excluded.

**Figure 4:**
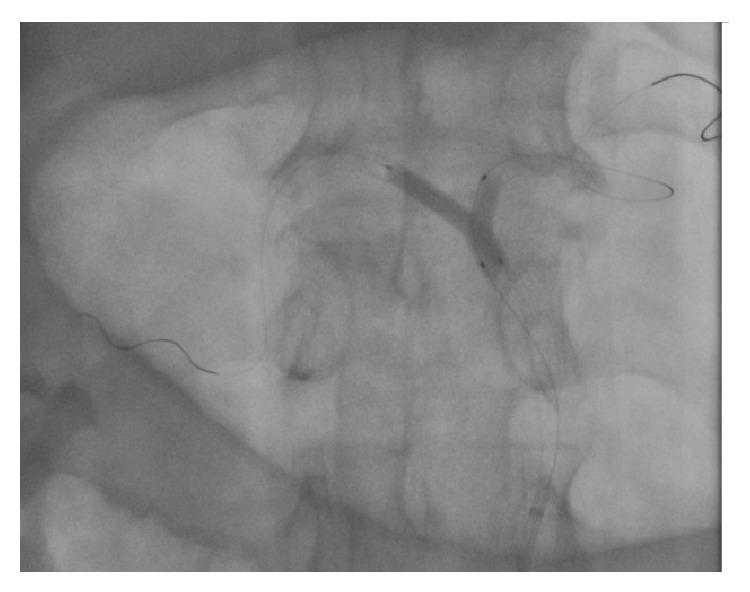
Final kissing.

**Figure 5:**
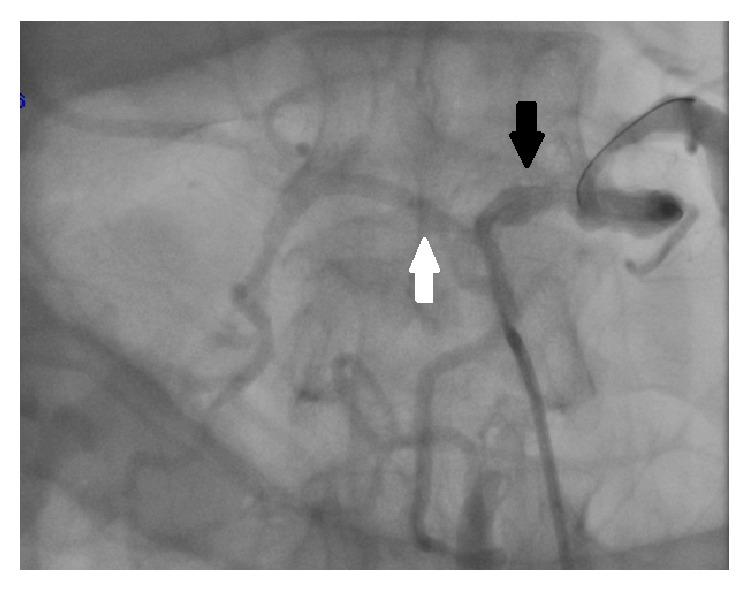
Postprocedure angiography showing good flow in HA (white arrow) and SA (black arrow).

The artery was predilated with 3 × 15 and 4 × 20 mm monorail balloons (Invader PTCA balloon, Alvimedica, Assen, Netherlands). A 4 × 24 mm balloon-expandable stent (Liberte Monorail Stent, Boston Scientific, MA, USA) was placed across the occlusion covering the ostium of the CA. An arteriogram confirmed an adequate flow to the splenic territory. A second 0.014-inch stiff guidewire (Asahi Standart, Asahi Intecc, Aichi, Japan) was able to cross the hepatic artery (HA) through stent struts. A 3 × 15 mm monorail balloon (Invader) was used to predilate the HA and expand the stent struts. Balloon dilatation did not establish an adequate flow within the hepatic artery. Therefore a stent implantation by utilizing TAP-stenting technique was decided in the CA bifurcation. Subsequently, the HA stent (Liberte Monorail 4 × 24 mm) was placed with minimal protruding into CA with an uninflated SA stent balloon for final kissing (Figure 4). After HA stent deployment, the balloon of the HA stent was slightly pulled into the CA and final kissing was performed. Final angiogram showed excellent flow within the CA, SA, and HA (Figure 5). Patient was transferred back to intensive care unit of surgery. For prevention of stent thrombosis aspirin (100 mg/d) and clopidogrel (75 mg/d) were initiated. Laparotomy was not performed because the patient did not develop clinical signs of bowel ischemia after endovascular procedure. A follow-up angiogram was not planned as a result of improvement in his condition.

## 3. Discussion

Various strategies have been offered for handling bifurcation lesions, each with exclusive virtues and technical challenges. A unique method has not yet been demonstrated. Hence, the optimum strategy still remains debatable. TAP-stenting (T-stenting and small protrusion of side branch stent) technique is a modification of the conventional T-stenting which allows full coverage of bifurcated lesions and facilitates final kissing balloon. The first step is wiring the main vessel (MV) and side branch (SB) followed by MV stenting and kissing balloon. Subsequently the SB stent is placed with minimal protruding into MV with an uninflated MV balloon for final kissing. After SB stent deployment, the balloon of the SB stent is slightly pulled into the MV and final kissing is performed. The TAP technique is comparatively a novel scheme that is technically less challenging, secures the side branch ostium with complete coverage, and minimizes stent overlap. Whilst there is fair amount of data for other bifurcation strategies, the long-term clinical outcomes for TAP technique are restrained. Despite the limited data TAP technique is related with satisfactory clinical results with no episodes of certain and likely stent thrombosis [3].

Antecedently more than 50% of patients diagnosed with mesenteric ischemia underwent open surgery. Currently almost half of patients get treatment with EI and demonstrate encouraging results. The bowel resection encounters less frequently. In hospital mortality after mesenteric ischemia is less with EI compared to surgery in which overall morbidity is higher and length of hospital stay is longer [4]. The superior mesenteric artery was generally the principal objective for revascularization, but reports have demonstrated resolution of symptoms after isolated CA as well [5, 6].

## 4. Conclusion

EI is an alternative to surgery for the CA occlusion in selected patients who have no signs of progressed bowel ischemia. Early diagnosis followed by immediate EI with meticulous postprocedural supervision is central.
